# Genetic modification of the protozoan *Eimeria tenella* using the CRISPR/Cas9 system

**DOI:** 10.1186/s13567-020-00766-0

**Published:** 2020-03-11

**Authors:** Xinming Tang, Jingxia Suo, Lin Liang, Chunhui Duan, Dandan Hu, Xiaolong Gu, Yonglan Yu, Xianyong Liu, Shangjin Cui, Xun Suo

**Affiliations:** 1 Institute of Animal Science, Chinese Academy of Agricultural Sciences, Beijing, 100193 China; 2 Key Laboratory of Zoonosis of Ministry of Agriculture & National Animal Protozoa Laboratory, College of Veterinary Medicine, China Agricultural University, Beijing, 100193 China; 3 Beijing Scientific Observation and Experimental Station of Veterinary Drugs and Diagnostic Technology, Ministry of Agriculture, Beijing, 100193 China

## Abstract

*Eimeria tenella* has emerged as valuable model organism for studying the biology and immunology of protozoan parasites with the establishment of the reverse genetic manipulation platform. In this report, we described the application of CRISPR (clustered regularly interspaced short palindromic repeat)/Cas9 (endonuclease) system for efficient genetic editing in *E. tenella*, and showed that the CRISPR/Cas9 system mediates site-specific double-strand DNA breaks with a single guide RNA. Using this system, we successfully tagged the endogenous microneme protein 2 (EtMic2) by inserting the red fluorescent protein into the C-terminal of EtMic2. Our results extended the utility of the CRISPR/Cas9-mediated genetic modification system to *E. tenella*, and opened a new avenue for targeted investigation of gene functions in apicomplexan parasites.

## Introduction, methods, and results

Members of the *Eimeria* genus are principal causative agents of coccidiosis in a wide range of livestock and birds [1]. With its distinct biology and immunology, the *Eimeria* parasite also holds promise as a model organism for protozoan research. The establishment of transient transfection and stable selection protocols has greatly enhanced dissection of several biological and immunological properties in this parasite [2, 3]. Nevertheless, the absence of an effective gene editing system has left many questions about functions of *Eimeria*’s unique genes unanswered.

The prokaryotic CRISPR (clustered regularly interspaced short palindromic repeat)/Cas9 (endonuclease Cas9) system is a recently developed tool for genetic editing in a variety of organisms [4, 5], including protozoan parasites *Toxoplasma*, *Plasmodium*, and *Trypanosoma* [6–9]. Due to lack of efficient in vitro tissue culture model and an effective selection marker, the technical capability for genetic modification of *Eimeria* has lagged behind for that of *Toxoplasma* and *Plasmodium*. The CRISPR/Cas9 system introduces site-specific double-strand DNA breaks (DSBs) with endonuclease Cas9 in a target sequence that is homologous to the single guide RNA (sgRNA). Subsequent repair of the DSBs is carried out by the host cell through either non-homologous end-joining pathway (NHEJ), leading to insertion and deletion mutations in the targeted genes, or homologous direct repair (HDR) in the presence of a DNA donor template. In this study, we present experimental evidence that the CRISPR/Cas9 system is also useful for targeted gene modification in *Eimeria*.

**Figure 1:**
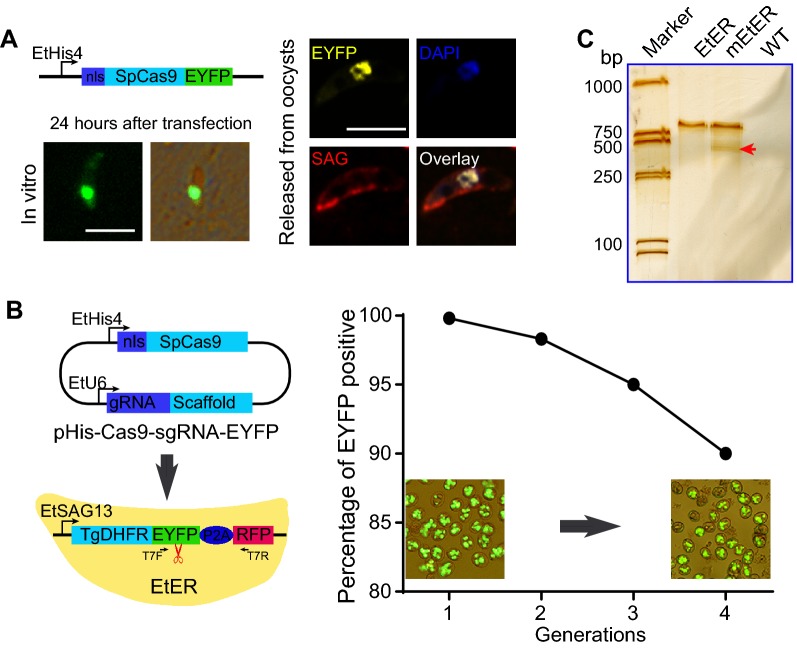
**CRISPR/Cas9 mediated target gene DSBs. A** SpCas9 expression was concentrated in the nucleus. The expression of SpCas9, which fused with EYFP, was controlled by EtHis4 promoter and nls as the schematic of the plasmid. Wild-type (WT) *E. tenella* sporozoites were transfected with the circular plasmid and then cultured in vitro or inoculated into the chicks for in vivo propagation. The EYFP signal was located in the nucleus of the sporozoites, both after in vitro culture and in vivo propagation (sporozoites released from the sporulated oocyst was stained with DAPI and polyclonal antibodies against SAG13 as index of parasites nucleus and periphery). Bar = 5 μm. **B** CRISPR/Cas9 mediated *eyfp* disruption. The SpCas9 expression and sgRNA (specific to *eyfp*) production, which were controlled by His4 and EtU6 promoter, were constructed in one plasmid. EtER sporozoites were transfected with this circular plasmid and then inoculated into the chicks for in vivo propagation. The ratio of fluorescence positive oocysts was decreased in the progeny of mEtER after adverse selection by FACS and propagation. **C** Mismatch repair of *eyfp* on the genome of mEtER was analyzed by T7 endonuclease I assay. Specific bands of original (863 bp) and mismatch repair (red arrow, ~430 bp) was detected after the visualization of mEtER. The genomic DNA of EtER and WT serve as control. Scissor logo: homologous sequence to sgRNA on the genome.

A circular plasmid with nlsSpCas9-EYFP expression cassette was constructed (Figure 1A), with the nlsSpCas9-EYFP driven by histone protein 4 (His4) promoter. The nuclear localization sequence (nls) of His4 was introduced into the N-terminal of *Streptococcus pyogenes* (SpCas9) to target the nucleus of the endonuclease SpCas9. SpCas9 was also tagged with enhanced yellow fluorescent protein (EYFP) gene for visualization of the SpCas9 expression signal in subcellular sites of *Eimeria*. Using this strategy, we found that the proportion of EYFP positive sporozoites was approximately 1.0 × 10^−3^ 24 h after transient transfection with the circular plasmid and in vitro culture according to the previous methods [10, 11]. The signals of EYFP and DAPI (4′,6-diamidino-2-phenylindole) were co-localized in the nucleus of sporozoites (Figure 1A). These results demonstrated that the nls of His4 efficiently localized SpCas9 to the nucleus.

RNA polymerase III promoters, such as human U6 promoter, have been widely utilized to drive guide RNA expression in eukaryotes [12]. We identified two RNA polymerase III U6 promoter candidates (EtU6-1 and EtU6-2; Additional file 1) in *E. tenella* genome by alignment with *Plasmodium* (the genomic location of EtU6-1 and EtU6-2 were HG673812: 317 896..318 493 and HG673812: 318 728..319 328 in ToxoDB, respectively). To confirm the site-specific DSBs by SpCas9 in a target sequence that is homologous to sgRNA in *E. tenella*, we conducted a “function lost” experiment of the target gene (EYFP). In brief, we constructed a double cassette plasmid (pHis-Cas9-sgRNA-EYFP): one is SpCas9 expression cassette regulated by His4 promoter and nls, the other is EYFP specific gRNA (5′-GCATGTGATCGCGCTTCTCGT-3′) cassette driven by EtU6-1 promoter (Figure 1B). The EtER sporozoites (1.0 × 10^7^), a recombinant *E. tenella* line with stable expression of EYFP and red fluorescent protein (RFP) [13], were transfected with circular plasmid of pHis-Cas9-sgRNA-EYFP (10.0 μg) using the AMAXA nucleofection system (program U033) and then inoculated to the chicks (each bird receiving a total number of 1.0 × 10^6^ transfected sporozoites) via the cloacal route. We found that the EYFP expression was lost with a ratio of 1.2 × 10^−3^ in the first-generation progeny of sporulated oocysts (Figure 1B). We propagated the population (EtER mutation, mEtER) after adverse selection, selected fluorescence negative population by fluorescence-activated cell sorting (FACS). The EYFP positive oocysts of mEtER was reduced to 89.5% after four generations’ selection (Figure 1B). These results suggested that CRISPR/Cas9 mediated site-specific DSBs of *eyfp* are repaired by NHEJ in the absence of donor DNA template. To confirm this observation, a T7 endonuclease I (T7EI) assay was performed to detect the mutation of *eyfp* after endogenous cleavages [14]. As T7EI recognizes and cleaves mismatched DNA, cruciform DNA structures, Holliday structures or junctions, heteroduplex DNA, and nicked double-stranded DNA, it enables detection of Cas9 induced mutations. In this study, genomic DNA of mEtER or EtER was amplified with T7F (5′-AGCAGCACGACTTCTTCAAGTCC-3′) and T7R (5′-CTTGGAGCCGTACTGGAACTG-3′) primers (Figure 1B). The PCR product (300 ng/sample) was denatured, reannealed, and digested with T7 endonuclease I (New England BioLabs) following the manufacturer’s instructions, which cleaves mismatched heteroduplex DNA. The reaction was analyzed by denatured PAGE. We detected a specific band of *eyfp* cleavage after silver staining in the sample of mEtER (Figure 1C). These results demonstrated that SpCas9 expressed by *E. tenella* efficiently mediated targeted gene cleavage guided by sgRNA, and that EtU6-1 promoter efficiently driven sgRNA production.

**Figure 2:**
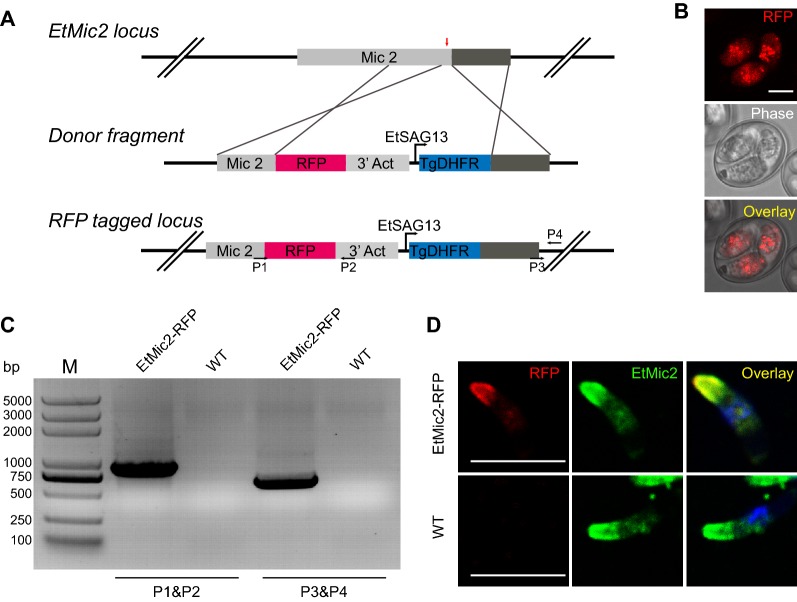
**CRISPR/Cas9 mediated endogenous gene tagging with a fluorescent protein. A** Schematic illustration for tagging EtMic2 with RFP. Donor fragment for HDR of DSB targets the EtMic2 C-terminal part of the coding sequence (CDS) (red arrow). The left arm (650 bp) is a part of EtMic2 CDS, and the right arm (527 bp) is the 3′ untranslated region of EtMic2. The directions and positions of primers P1 to P4 for PCR identification are indicated by black arrows. **B** RFP positive sporulated oocysts were observed in 1st generation of EtMic2-RFP. Bar = 5 μm. **C** Diagnostic PCR demonstrates homologous integration in EtMic2-RFP compared with WT. Predicted size products (808 and 699 bp) were detected after PCR amplification using P1 (5′-CCCTTGATTGCTGTTCGCATCCAT-3′) and P2 (5′-GATCTCGAAGTAGTGGCCGTTCAC-3′) or P3 (5′-GCCGAGGATTTTGAGGTCGTGG-3′) and P4 (5′-TTACCCATGTGGAAGCAACATTGG-3′) primers (**A**) from genomic DNA of EtMic2-RFP. **D** Co-localization of the RFP and EtMic2 EtMic2-RFP sporozoites. EtMic2-RFP sporozoites of the 3rd generation were stained with mouse anti-EtMic2 monoclonal antibody and subsequent reaction with FITC-conjugated goat anti-mouse IgG (Proteintech Group). The sporozoites were visualized using a confocal laser scanning microscopy (SP5, Leica, Germany). Sporozoites from wild type (WT) *E. tenella* serve as control. Bar = 5 μm.

Next, we tagged microneme protein 2 (EtMic2) with a red fluorescent protein (RFP) using CRISPR/Cas9 system to investigate whether this system could mediate endogenous gene modification in *Eimeria*. In this experiment, a donor fragment and a SpCas9 and sgRNA (target at 3′ terminal of EtMic2 gene, sequence information: 5′-GGCGTCTCGATTGTGAGAGC-3′) producing plasmids were constructed. The following four DNA fragments: left homologous arm, *rfp*-3′Act [11], EtSAG13 promoter-TgDHFR [13], and right homologous arm were ligated according to the instruction of HiFi DNA Assembly Master Mix kit (New England BioLabs) to generate the donor fragment (Figure 2A; Additional file 1). The *E. tenella* sporozoites (1.0 × 10^7^) were co-transfected with the donor fragment (10.0 μg) and the circular plasmid (2.0 μg), which produce SpCas9 and sgRNA. The transfected sporozoites were inoculated into five 1-week-old chicks via the cloacal route. The chicks were fed with pyrimethamine (150 mg/kg) containing diet. We detected RFP positive oocysts with a ratio of 2.1 × 10^−4^ in the progeny of the first generation (Figure 2B). The RFP positive population (EtMic2-RFP) was increased by 25.8% after three generations’ selection with FACS and pyrimethamine. To determine if the EtMic2 are accurately tagged with RFP as predicted, PCR and indirect immunofluorescent assay (IFA) [15] were performed. We found specific bands after PCR amplification from the genomic DNA of EtMic2-RFP (Figure 2C). And the RFP and EtMic2 were perfectly colocalized in the microneme of EtMic2-RFP sporozoites (Figure 2D). These results demonstrated that the CRISPR/Cas9 system efficiently mediates target gene break and repair by HDR and that the system mediates endogenous gene modification in *Eimeria.*

## Discussion

Exhibiting distinct biology and immunology, *Eimeria* represents a model organism for experimental study of apicomplexan parasites. Although reverse genetic technologies for the *Eimeria* parasite are well developed [10, 11, 16], the targeting of specific loci is limited by the fact that recombination tends to occur by nonhomologous integration of foreign DNA into the chromosome in apicomplexan parasites [17, 18]. This makes it desirable to develop an alternative method for precise gene targeting in *Eimeria*.

The prokaryotic CRISPR/Cas9-based genome editing system is a versatile, widely adopted tool for genetic editing in biological organisms, including *Caenorhabditis elegans* [19], zebrafish [20], and mammalian cell [21]. The CRISPR/Cas9 system relies on endonuclease Cas9 expression in the nucleus and spatial conformation of sgRNA for producing site-specific DSBs. As shown in this report, with the capability to knock-out (KO) and knock-in (KI) *E. tenella* genes (e.g., EYFP gene disruption and EtMic2 tagging with RFP), the CRISPR/Cas9 system offers a valuable approach for genetic manipulation and functional study of apicomplexan parasites.

While the two EtU6 promoters, EtU6-1 and EtU6-2, are capable of driving sgRNA production in the primary study (data not shown), there is no significant difference in efficiency between the two promoters. So, only EtU6-1 was examined here to reduce unnecessary repetitive experiments. As shown above, nls of His4 and U6 promoters (i.e., EtU6-1 promoter) of *E. tenella* readily localize SpCas9 to the nucleus and produce sgRNA (Figure 1B), and the DSBs could be repaired by HDR in the presence of a DNA donor template (Figure 2), despite the fact that the efficacy in *Eimeria* appears much lower than that in *T. gondii* (~20 to 30%) or *Plasmodium* spp. (~20%) [7, 8]. Low transfection (especially co-transfection) efficiency may be one of the reasons for the low genetic editing efficacy of *Eimeria* parasite using the CRISPR/Cas9 system. Therefore, further optimization of the transfection system is crucial for improving the effectiveness of gene modification in *Eimeria* [22].

Most of the selectable marker genes used for *T. gondii* are unsuitable for selection of stable transformants in *Eimeria*, owing to the following restrictions, including: (1) the cost of the reagent, like rapamycin analog Shield-1, is prohibitive for identifying stable transfection of *Eimeria* in an in vivo selection system; and (2) some selectable marker genes only work in specific recipient strains of *Toxoplasma*, like TATi and *hxgprt*-. Despite these restrictions, FACS and pyrimethamine selection protocols have been successfully developed for *Eimeria*. Further refinement of selection system is clearly imperative for improved genetic manipulation of *Eimeria*.

NHEJ is the preferred mechanism for insertion of foreign DNA into the genome of *T. gondii*, not homologous integration [23]. No direct evidence is available yet as *Eimeria*’s preference for insertion of foreign DNA (NHEJ or homologous integration). In view of previous findings that foreign DNA with ~1000 bp homologous fragments are randomly inserted at different sites in the genome of recombinant *Eimeria* [11, 13], and current evidence of precise integration of foreign DNA, NHEJ may be the preferred mechanism for insertion of foreign DNA into the genome of *Eimeria* just like *Toxoplasma*. To further enhance the efficient in genetic editing of *Eimeria*, inactivation of the NHEJ pathway, such as knocking out the critical component KU80 will likely increase the efficiency of homologous recombination, thus improving genetic editing in *Eimeria*.

In conclusion, we successfully developed the CRISPR/Cas9 system in *Eimeria,* which will accelerate gene function studies in *Eimeria* and apicomplexan parasites.

## Supplementary information

**Additional file 1. Sequences of DNA fragments in this study.** Nucleotide sequences of EtU6 promoters and donor fragment for EtMic2 tagging were listed in this file.

## Abbreviations

CRISPR: clustered regularly interspaced short palindromic repeat
DSBs: double-strand DNA breaks
sgRNA: single guide RNA
HDR: homologous direct repair
SpCas9: *Streptococcus pyogenes* endonuclease Cas9
nls: nuclear localization sequence
His4: histone protein 4
EYFP: enhanced yellow fluorescent protein
RFP: red fluorescent protein
EtMic2: microneme protein 2 of *E. tenella*
EtER: a transgenic *E. tenella* line expressing EYFP and RFP
FACS: fluorescence-activated cell sorting
CDS: coding sequence
KO: knock-out
KI: knock-in
