# Supplementary material for: Genetic modification of the protozoan Eimeria tenella using the CRISPR/Cas9 system
Source: Vet Res. 2020 Mar 11;51:41. doi: 10.1186/s13567-020-00766-0 (PMC7065449; doi:10.1186/s13567-020-00766-0)
Supplement: Supplementary file 1 — Additional file 1. Sequences of DNA fragments in this study. Nucleotide sequences of EtU6 promoters and donor fragment for EtMic2 tagging were listed in this file. [file 13567_2020_766_MOESM1_ESM.docx]

**Additional file 1 Sequences of DNA fragments and plasmids in this study.**

1. **Sequences of EtU6-1 and EtU6-2 promoter**

EtU6-1 (HG673812: 317,896..318,493):

GCTGCAGTGCGTTGCAAGAAGTGCATAATATTTTCTTTTATTGGCCAAGCTGTGGGTCCTTCACTCCGCCTTGTTGTCCTGCCGCAGGCCTTTCTGTAAAGAATGTACTGCCCTGTCCCGTCGACTCCTGGATATGCATGTTTTGAATTGGAAAATTGCAGTTTGGTGCTTTTTTTCGTTTGAAATCCTTTTTCCGGCCTCAACTGGTTGTTCACACGTTCGACTCCCTCTGGTGCGGCTTCATGGTCCACAAGGAAACCATATTCTAAGTAGGGAGGTAGAGGAATGAGCCTTTCACATCTGAATATGACTTAAAGCTTCACTTGTCCATTTATTCCAATAACATAAAGTACAAAAGCAACTCTCCCACCAGGTCTTCCAACCCATCTCGGCAGTCACGTTCGCCCCAGCAGCAGAAACCCGCTCGTCTCGTATATCACACAAGCGGATTAGATCTTGAAAAATATTTGTTATTTTTGCCTTTTAAACATTCAAAGAATGTTATTCAAGGCAAAAGAACGGAAAGGCGGTCATAGTGTCTCAGGTGACCTCACCCCGTGATGGGTGTTCCTTCTGTACGAGAGTGGGTTAAGAGGAT

EtU6-2 (HG673812: 318,728..319,328):

GTCATTTAGTTGGTAGCTGCTACTTTGTGACACTTTGACACCGTTAGAGACTCAGAGCTTGAGGATCTCATCAAGGATCAGTTCGCGTCCAAACGCACTGTCCACCCGAGTCATGCGTCGTGTCACGGCGGAGCCTCTTTTTGATGTTTTTAATATGAATGTTAAGTGTTACAATGCACCAAGATGGTTCTACGGTTCATTGGTACATCTCGTCGAAGTAAAGGCCACTCTACTTTCGTGCCACCCAGGTCGCCAGATCTCCCTTCTCTCCTACACCTCATATTGCCCTTAACCTCTCAAATCTCATTCCCGTCAAAACAATCTTGATATCCGAGTCAAGTAGTGCCTCCTGTAAGTCGTGGCTCCTCTCCACCTCCCCTCTAGCTGTTGCGCAGCGTAGAGCTGTTCAAGCATCTCTCCTCACTGTTGTTGCAAACTAGCAAGCGGATGTGGCAAAAAAGCACACAATATATGAATATAATCGAAAAAAAATTGGAGAAAATTGGTGGAAACGTGGAAGTGCTACTGGGAGTCGATGCCGCAGTGAGGTGACCCTGTACGCGATGGGCGTTCCTTTAATACGAAGACGGCACTTCAGGTT

1. **Sequence of donor fragment for EtMic2 tagging:**

CGGAAATGATGAAAGCGAGCCTACGGAGGTTCCCCTAGAAACAGCAGCTGGACCGACCACGCCACTCATGGTACTCATTACGCAGCAGAACCCAAAGGAAGTGGAAGTCCGTGTTCTTGCTTGGATATCTACGTAAGTTCACCCCCTTTGAGTTGCAGCGTCGTTCTGGTGTATATTCGTTGGCAAGTGAATATTTGTGTAGCCGGAGAGGCGTTTCGCGGATCTTCAAGTATAGTGACTTCTGTTCTCTCTGCAGGGACGCTACAACTGGAAAGGGCTCTTGGAAAGAAAATTCCGTGGTCGTTGGCAGCTCCTTGAGCGGGCGCGACCTTACCGTGAACTTGAGCGACTGTGGACCAAGCTCCCTCAGGGTTTATGGCTCGGCATCAGCTGACCTTGTAACTGTCAAGGAGGGCATGTGTGAGGCAGACGACCCAGAGTTGATCGCGCTGACTCGGCCTCATACATCGGCAGCTTCTCCGCTGCCTGCAGAGGAAGGAGACGTAGCGCAGGACGCCCAGCAGAGCGCAGGAGCCCAGCAGGAAGCAGAAGCCCAGGAGGTTGGAGAACCCCAGCAGGAAGCAGCTGCTGCAGAGCAAGGAAGCAGCGCTGCAGAGAGTGACACTCAACAGTCATCCATGGACAACACCGAGGACGTCATCAAGGAGTTCATGCAGTTCAAGGTGCGCATGGAGGGCTCCGTGAACGGCCACTACTTCGAGATCGAGGGCGAGGGCGAGGGCAAGCCCTACGAGGGCACCCAGACCGCCAAGCTGCAGGTGACCAAGGGCGGCCCCCTGCCCTTCGCCTGGGACATCCTGTCCCCCCAGTTCCAGTACGGCTCCAAGGCCTACGTGAAGCACCCCGCCGACATCCCCGACTACATGAAGCTGTCCTTCCCCGAGGGCTTCACCTGGGAGCGCTCCATGAACTTCGAGGACGGCGGCGTGGTGGAGGTGCAGCAGGACTCCTCCCTGCAGGACGGCACCTTCATCTACAAGGTGAAGTTCAAGGGCGTGAACTTCCCCGCCGACGGCCCCGTAATGCAGAAGAAGACTGCCGGCTGGGAGCCCTCCACCGAGAAGCTGTACCCCCAGGACGGCGTGCTGAAGGGCGAGATCTCCCACGCCCTGAAGCTGAAGGACGGCGGCCACTACACCTGCGACTTCAAGACCGTGTACAAGGCCAAGAAGCCCGTGCAGCTGCCCGGCAACCACTACGTGGACTCCAAGCTGGACATCACCAACCACAACGAGGACTACACCGTGGTGGAGCAGTACGAGCACGCCGAGGCCCGCCACTCCGGCTCCCAGTGAGTTTGCAGCAGAGTAGTTCATGACTTGCGAAACGGCCTCTCGATTAATAATACGCTTTGCCGCAACGTGAAGTAGGCGTTATTGTGTGCTGCCTGTCGCTGAGCTCCTGCATCGAGCGGCAAGGGGTTCAACCGAGCGCAAATTCTGTGGAAATAGCTGGACAAAAGCATCTGCGACGGGTGGGGGCAGGCGCAGCTAGCTGTTGCTCCCGACATCAGTCATGGAAATGCGTTTCAGGCTAAGCAAGTAGCTGCCCGCCTGGTGGGAAGATGAGGCTAGGAACTGCTATGTTTGCCCCTCACTTGGCGGTATTGCAGTGTAGTACGTGCATTGTGAACCCAGAGAAATGTGCTCCGCGTGACGCAGCGAGGCACAGGGGAACAGCAAGAGGGGCCGTTATTGCTCAGCGTGCTGGACCCCCTTGTTCCTTCAAACCTTACTTATGAGGGCTTACGCATCGCACCTGATGGGTCGGTGTAGCGTGATCATTTCACTGTTCAGTAGTAGGTATGGGAGGAGTGTAGTTGGCAGGATGTAGGAGCTTTGCAAGCGGTGGAACGGTTGAGATGAGTAGTGAACAGAGGCTTTGCCCAATGTCGGATAGTGAGTGCCTAGGATAACTTCGTATAGCATACATTATACGAAGTTATTGTATCATATTAATTCCGGCCAGTGTCTCTAAATAAACTCTTTTGTAGGATCTGCCAACTCACTGCGGGGGGGCACACAGAAGCACCACATTGCACGACACTGCTTTTGCGCAGGCTGCGGGGCAATTAAACGTTAACAGTGCTTGATCCGGCCGAAATGACGCGAAATATTTAAAACATTATCTGTTTGTCAGCCTAACGGAGATGACATGGTCGCACAAGAGACTACAGTGTGTGTGTGATGTCTTTCGTGCATCCCACCGAGCCTCTGGAATTCGGCACCGCATTAGCCCACACGTAAAACATTGCGTACCTAACCAAGAAGACTTCTCGGGCAGAGAAAATGTAAAATTATACATTAGCAGAGCCACACATTACACTAAATTGTTTAATTAGGCTTGGTTCACGTTCCGCAGCTCGTAATGCGGGCGCGCGACAGATCAACACACACACACAAGCATCTCGGCAGCACGTAGTCACTAGTGGAATAACCGCGCACTTCAACAGACCATTAAAAACCTAGACATTTATATCCTAAAGCATGTTCTGTTGAAGCTTCACAAAGCAGAATTCTAAGACAGCTTAGCTCGTCGCAACTCAGGTGGTGAAACAGCACCATGCTCTAGCTGGCAGCTGGGACTGTCACGTCGATGAGCCCTGAGTTTCTCATCGTACACACGCATTTTTCCCAGCATTTCAATTTTTTTTGTTGACCCGGGTGTGCTCGCCCACTTTGTTCCTGTTGTCCCTTTGCTTTCTGTGTTTTTCCGCAGCGGCCGCATGCAGAAGCCGGTGTGTCTGGTCGTCGCGATGACCCCCAAGAGGGGCATCGGCATCAACAACGGCCTCCCGTGGCCCCACTTGACCACAGATTTCAAACACTTTCGTCGTGTGACAAAAACGACGCCCGAAGAAGCCAGTCGCCTGAACGGGTGGCTTCCCAGGAAATTTGCAAAGACGGGCGACTCTGGACTTCCCTCTCCATCAGTCGGCAAGAGATTCAACGCCGTTGTCATGGGACGGAAAAACTGGGAAAGCATGCCTCGAAAGTTTAGACCCCTCGTGGACAGATTGAACATCGTCGTTTCCTCTTCCCTCAAAGAAGAAGACATTGCGGCGGAGAAGCCTCAAGCTGAAGGCCAGCAGCGCGTCCGAGTCTGTGCTTCACTCCCAGCAGCTCTCAGCCTTCTGGAGGAAGAGTACAAGGATTCTGTCGACCAGATTTTTGTCGTGGGAGGAGCGGGACTGTACGAGGCAGCGCTGTCTCTGGGCGTTGCCTCTCACCTGTACATCACGCGTGTAGCCCGCGAGTTTCCGTGCGACGTTTTCTTCCCTGCGTTCCCCGGAGATGACATTCTTTCAAACAAATCAACTGCTGCGCAGGCTGCAGCTCCTGCCGAGTCTGTGTTCGTTCCCTTTTGTCCGGAGCTCGGAAGAGAGAAGGACAATGAAGCGACGTATCGACCCATCTTCATTTCCAAGACCTTCTCAGACAACGGCGTACCCTACGACTTTGTGGTTCTCGAGAAGAGAAGGAAGACTGACGACGCAGCCACTGCGGAACCGAGCAACGCAATGAGCTCCTTGACGTCCACGAGGGAGACAACTCCCGTGCACGGGTTGCAGGCTCCTTCTTCGGCCGCAGCCATTGCCCCGGTGTTGGCGTGGATGGACGAAGAAGACCGGAAAAAACGCGAGCAAAAGGAACTGATTCGGGCCGTTCCGCATGTTCACTTTAGAGGCCATGAAGAATTCCAGTACCTTGATCTCATTGCCGACATTATTAACAATGGAAGGACAATGGATGACCGAACGGGCGTTGGTGTCATCTCCAAATTCGGCTGCACTATGCGCTACTCGCTGGATCAGGCCTTTCCACTTCTCACCACAAAGCGTGTGTTCTGGAAAGGGGTCCTCGAAGAGTTGCTGTGGTTCATTCGCGGCGACACGAACGCAAACCATCTTTCTGAGAAGGGCGTGAAGATCTGGGACAAGAATGTGACACGCGAGTTCCTCGATTCGCGCAATCTCCCCCACCGAGAGGTCGGAGACATCGGCCCGGGCTACGGCTTCCAGTGGAGACACTTCGGCGCGGCATACAAAGACATGCACACAGACTACACAGGGCAGGGCGTCGACCAGCTGAAGAATGTGATCCAGATGCTGAGAACGAATCCAACAGATCGTCGCATGCTCATGACTGCCTGGAATCCTGCAGCGCTGGACGAAATGGCGCTGCCGCCTTGTCACTTGTTGTGCCAGTTCTACGTGAACGACCAGAAGGAGCTGTCGTGCATCATGTATCAGCGGTCGTGCGATGTCGGCCTCGGCGTCCCCTTCAACATCGCTTCCTATTCGCTTTTGACGCTCATGGTTGCACACGTCTGCAACCTAAAACCTAAGGAGTTCATTCACTTCATGGGGAACACGCATGTCTACACGAACCATGTCGAGGCTTTAAAAGAGCAGCTGCGGAGAGAACCGAGACCGTTCCCCATTGTGAACATCCTCAACAAGGAACGCATCAAGGAAATCGACGATTTCACCGCCGAGGATTTTGAGGTCGTGGGCTACGTCCCGCACGGACGAATCCAGATGGAGATGGCTGTCTTGGATCCTGTCGCCACCTGAAGGACTGCTTAAAAATGTGCAGTGTTGATCTGGAAGAGGTTCAAGCAGAGTGACGCAAAGACGTGTCGCTTGCTTTGATGTTGGCTTCCGGAGACGAATGCATGCATTATTTTGGCATTTTCCCATTCGCGGAGCAGCGGCTTACAGGAGGATGACACGGCTTTCATTGTGCTTCATGACGCCCACTGTTACACACCTTCGCCATTGCTCTTTTTGGAATTTGGGTGTCCGGCATTTTTTTGGCCACACAAACGCACTTCCTCACATCCACCCAGCCACATGTACACATGTACACAAACACATGCAAACACAACGTGCATTCACTGTGAAGTTGCCGTGTGGCAGCGCTCACGGGGGGAAGGTAGCGGGGGAAAGGCAGGTCCCCCGCAGCGGTAGATGCAGGTTTATTCTACGGCAGGTAGAGGTTGCAGCGAATGGGGGATTCGTTTGCTGAGTAAATTTCATGCAGGTCAGTGCCTTACTAGAACATGAATATGGTCCCACAGAGAGCTTGTTGTAAGCTATGG

Note:

Left homologous arm: 1-638

RFP: 639-1316

3’ UTR (Actin): 1317-1927

EtSAG13 promoter: 1928-2746

TgDHFR: 2747-4597

Right homologous arm (3’ UTR): 4598-5124
